# Osteoprotegerin, Chitinase 3-like Protein 1, and Cardiotrophin-1 as Potential Biomarkers of Obstructive Sleep Apnea in Adults—A Case-Control Study

**DOI:** 10.3390/ijms24032607

**Published:** 2023-01-30

**Authors:** Piotr Fiedorczuk, Ewa Olszewska, Joanna Rogalska, Małgorzata M. Brzóska

**Affiliations:** 1 Department of Otolaryngology, Medical University of Bialystok, 15-089 Bialystok, Poland; 2 Department of Toxicology, Medical University of Bialystok, 15-089 Bialystok, Poland

**Keywords:** obstructive sleep apnea, biomarkers, plasma and serum concentrations

## Abstract

Obstructive sleep apnea (OSA) is a prevalent, underdiagnosed disease and is considered a risk factor for cardiovascular diseases, depression, accidents, and stroke. Recent clinical practice guidelines for OSA expressed the need for a new clinical tool that establishes the Apnea–Hypopnea Index (AHI) to determine the disease burden. The serum and plasma concentrations of Osteoprotegerin (OPG), Chitinase 3-like protein 1 (YKL-40), and Cardiotrophin-1 (CT-1) in 80 subjects—52 OSA patients, 27 moderate (15 ≤ AHI ˂ 30) and 25 severe (AHI ≥ 30), and 28 non-OSA controls (AHI 0–5)—were determined. Moreover, the Total Oxidative Status (TOS), Total Antioxidative Status (TAS), and Oxidative Stress Index (OSI) were assessed in the serum and plasma to evaluate whether the severity of OSA and the concentrations of OPG, YKL-40, and CT-1 correlate with the oxidative/reductive status. The serum and plasma concentrations of YKL-40 and CT-1 were higher in the OSA group, whereas the serum and plasma concentrations of OPG were lower compared to the control group. The concentrations of OPG, YKL-40, and CT-1 in the serum and plasma correlated with AHI; however, a better correlation of the concentrations was obtained for the above-mentioned proteins in the plasma. The concentrations of YKL-40 and CT-1 in the serum and OPG in the plasma show better diagnostic capabilities for moderate and severe OSA than the concentrations of YKL-40 and CT-1 in the plasma and the concentrations of OPG in the serum.

## 1. Introduction

Obstructive Sleep Apnea Syndrome (OSA) is a disorder with a high prevalence, estimated at 9% to 38% in the general adult population, 13–33% in men, and 6–19% in women [1]. Up to 90% of individuals with OSA remain without a diagnosis or therapy. Patients with untreated OSA are at increased risk of hypertension, cardiovascular disease, heart failure, obesity, metabolic dysregulation, diabetes mellitus, daytime sleepiness, depression, accidents, and stroke [2,3,4,5]. The pathogenesis of OSA is multifactorial and still not fully established. It involves various mechanisms, including selective activation of inflammatory molecular pathways, endothelial dysfunction, metabolic dysregulation, and oxidative stress [6,7]. Endothelial dysfunction is often considered one of the earliest detectable and possibly reversible abnormalities during the development of atherosclerosis [8].

Cardiovascular and pulmonary complications are thought to be caused by tissue injury associated with chronic intermittent hypoxia [9]. The mechanisms of the injury of these tissues are not known. OSA stimulates several mechanisms, including inflammation, endothelial dysfunction, and oxidative stress, mainly through intermittent hypoxia. In intermittent hypoxia, the inflammatory signaling factors play essential roles in the transcriptional regulation of inflammatory cytokines [10]. However, their role in OSA pathogenesis and the exact relationships between pathogenic mechanisms remain unclear. Oxidative stress and a chronic inflammatory state are the main characteristics of the pathophysiological changes in OSA, contributing in consequence to the development of neural, cardiovascular, and metabolic alterations [11].

Recent clinical practice guidelines for OSA expressed the need for a new clinical tool to determine the disease burden [2]. New methods of screening and diagnosis, other than an overnight sleep study, to establish the Apnea–Hypopnea Index (AHI) may provide an easier diagnostic process to identify patients with OSA and allow earlier dispense of treatment, e.g., positive airway pressure—PAP therapy, thus preventing serious comorbidities. Perhaps the measurement of the concentrations of the serum or plasma inflammatory cytokines, markers of the dysfunction of the endothelium, and proteins associated with intermittent hypoxia can aid in OSA screening, prognosis, diagnosis, and monitoring of the effectiveness of treatment [12]. Taking into account available literature data, it is feasible that Tumor Necrosis Factor Receptor Superfamily Member 11b (TNFRSF11B), also called Osteoprotegerin (OPG), Chitinase 3-like protein-1 (YKL-40), and Cardiotrophin-1 (CT-1) could become candidates for potential biomarkers of OSA.

OPG is a glycoprotein, produced by osteoblasts, B lymphocytes, monocytes, fibroblasts, and vascular smooth muscle cells, that is thought to promote and induce endothelial cell dysfunction, vascular inflammation, and atherosclerosis [13]. The relationship between OPG and OSA remains inconclusive. Patients with OSA had substantially increased concentrations of OPG in the plasma [14]. After controlling for confounding variables, such as age, sex, body mass index (BMI), presence or absence of OSA, coronary heart disease, diabetes mellitus, hypertension, uric acid, creatinine, and C-reactive protein, the concentrations of OPG in the plasma were found to correlate only with age and with the occurrence of OSA.

YKL-40 is a 40 kDa glycoprotein. It is released by several types of innate immune system cells, including endothelial cells, epithelial cells, and smooth muscle cells [15]. Elevated concentrations of the serum or plasma YKL-40 were associated with OSA and other diseases, such as type 2 diabetes mellitus, atherosclerosis, endothelial dysfunction, and hypertension [16,17,18]. A 2019 meta-analysis by Zhang et al. found that OSA patients exhibit higher blood YKL-40 concentrations, which might be used as a biomarker for OSA diagnosis and screening [19]. The study expressed the need for more consistent and robust investigations to better understand the significance of serum concentrations of YKL-40 in OSA patients and to evaluate whether monitoring their concentrations can reflect disease phases and support successful therapeutic decisions.

CT-1 is a member of the family of cytokines that includes leukemia inhibitory factor (LIF), ciliary neurotrophic factor (CNTF), oncostatin M (OSM), interleukin-6 (IL6), and interleukin-11 (IL11), and it performs pleiotropic functions in various tissues [20]. In several conditions, including hypertensive heart disease, coronary artery disease, aortic stenosis, and dilated cardiomyopathy, CT-1 encourages myocardial structural alterations and participates in left ventricular remodeling, leading to left ventricular failure. CT-1 is defined as the key regulator of energy homeostasis [21]. Recent scientific research has shown that CT-1 may also regulate the body’s metabolic processes. CT-1 is considered a new indicator of inflammation in the form of a systemic inflammatory response. It was shown that the concentration of CT-1 in the serum was higher in patients with OSA compared to the control group without OSA, and based on these findings, it has been hypothesized that CT-1 may be an early biomarker for OSA detection [22]. Moreover, other studies reported no differences between OSA patients and non-OSA controls [23]. 

Most studies available to date reported the concentrations of OPG, YKL-40, or CT-1 in one cell-free blood fraction, serum, or plasma. During the coagulation process for serum collection, proteins and metabolites are released by platelets into the serum. Contrarily, plasma is initially treated with anticoagulants (such as EDTA, heparin, and citrate) before the blood cells are separated by centrifugation, ensuring that the liquid still contains deactivated clotting components. These processes can cause alterations in the metabolome or proteome and affect the concentrations of OPG, YKL-40, and CT-1 in the serum and plasma [24]. To our knowledge, no study attempted to compare the diagnostic capability of OPG, YKL-40, and CT-1 between the concentrations in the serum and plasma.

Inflammatory signaling factors are essential in the transcriptional control of inflammatory cytokines and the stimulation of oxidative stress in intermittent hypoxia during sleep [25]. In OSA, reactive oxygen species (ROS) generation by oxidant-producing systems and antioxidant defense mechanisms are out of balance, which results in oxidative stress [26]. Because oxidative stress is involved in the pathogenesis of OSA [11], Total Oxidative Status (TOS) and Total Antioxidative Status (TAS) were determined in the serum and plasma, and the Oxidative Stress Index (OSI) was calculated to evaluate whether these parameters correlate with the severity of OSA. Moreover, it was examined if there exists a dependence between the oxidative/reductive status and the concentrations of OPG, YKL-40, and CT-1 in the serum and plasma.

This study aimed to investigate whether OPG, YKL-40, and CT-1 may be useful in OSA diagnosis and differentiation of the severity of the disease. We compared the concentrations of serum and plasma OPG, YKL-40, and CT-1 in a single patient cohort, between moderate and severe OSA patients and non-OSA controls. We also evaluated if the concentrations of OPG, YKL-40, and CT-1 in the serum are divergent to the concentrations of OPG, YKL-40, and CT-1 in the plasma and assessed which have a higher predictive capability in diagnosing OSA.

## 2. Results

We enrolled a total of 80 subjects in our study, including 52 diagnosed with moderate (15 ≤ AHI ˂ 30) and severe (AHI ≥ 30) OSA and 28 non-OSA controls. Table 1 displays the demographic, anthropometric, and biological characteristics of the participants of the study.

In both the OSA and control groups, mostly male patients were enrolled. Subjects in both groups were matched in terms of age and sex, although the OSA group had a higher BMI compared to the control group. Subjects in the OSA group had higher AHI compared to the control group—this measurement was the basis of the allocation of the subjects to the groups. The AHI of all subjects correlated with BMI (*p* < 0.0001), but not with age (*p* = 0.1236). Patients from the OSA group were divided into two subgroups based on AHI—28 patients with AHI ≥ 15 but <30 into the Moderate OSA group and 25 patients with AHI ≥ 30 into the Severe OSA group. Both the OSA and control groups included patients suffering from hypertension, with a higher prevalence in the Severe OSA group. Other comorbidities, such as diabetes mellitus, coronary artery disease or hyperuricemia, and the percentage of patients who smoke were also reported.

### 2.1. OPG, YKL-40, and CT-1

The calculated medians, minimum-maximum values, and interquartile ranges of the concentrations of OPG, YKL-40, and CT-1 in the serum and plasma, and the effect size of calculations are demonstrated in Table 2. Subjects with OSA had a 2.3 times higher concentration of YKL-40 in the serum, a 3.7 times higher concentration of CT-1, and a 77% lower concentration of OPG compared to the non-OSA control group.

In the plasma, the concentration of YKL-40 was 2 times higher, CT-1 concentration was 2.6 times higher, and OPG concentration was 78% lower when comparing the OSA group to the control group.

The results of the analysis of correlation for all subjects, the control group, the Moderate OSA group, and the Severe OSA group are shown in Table 3. In the entire group, the concentrations of YKL-40 and CT-1 in the serum and plasma positively correlated with AHI, and the concentrations of OPG negatively correlated with AHI.

The correlation analysis between AHI and the concentrations of OPG, YKL-40, and CT-1 showed a higher Spearman correlation coefficient in the plasma than in the serum, in the combined OSA group and the control group. For the control group, the Moderate OSA group, and the Severe OSA group, the concentrations of YKL-40 in the plasma positively correlated with AHI in the control group, but not in the other subgroups.

**Figure 1:**
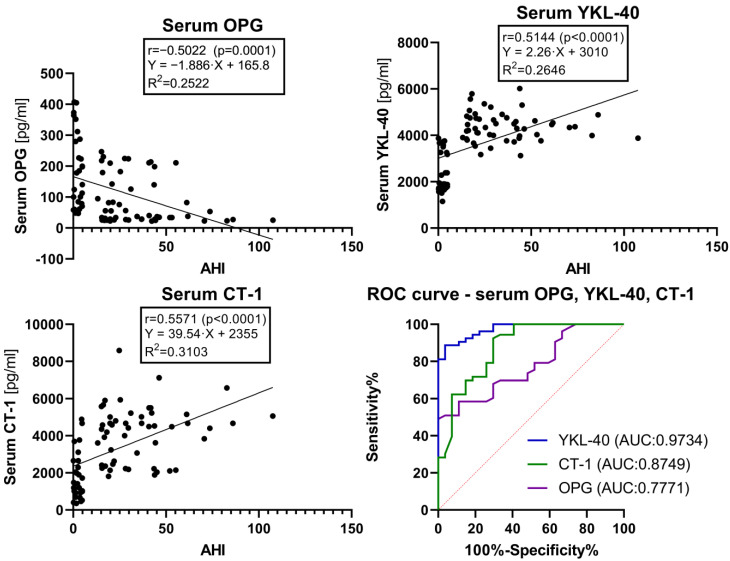
Simple linear regression graphs of the concentrations of OPG, YKL-40, and CT-1 in the serum. ROC curves of the concentrations of OPG, YKL-40, and CT-1 in the serum. The results of Spearman correlation analysis are expressed as r values and the level of statistical significance (*p*). The values of r with *p* < 0.05 were considered statistically significant. The coefficient of determination is expressed as R^2^. The simple linear regression models for serum OPG, YKL-40, and CT-1 use AHI as a single explanatory variable. The Receiver Operator Characteristic curve analysis is calculated for all subjects in classifying for OSA and non-OSA groups. Abbreviations: OSA, Obstructive Sleep Apnea; OPG, Osteoprotegerin; YKL-40, Chitinase-3 like protein-1; CT-1, Cardiotrophin-1; AHI, Apnea–Hypopnea Index; ROC, Receiver Operator Characteristic; AUC, Area Under the Curve.

**Figure 2:**
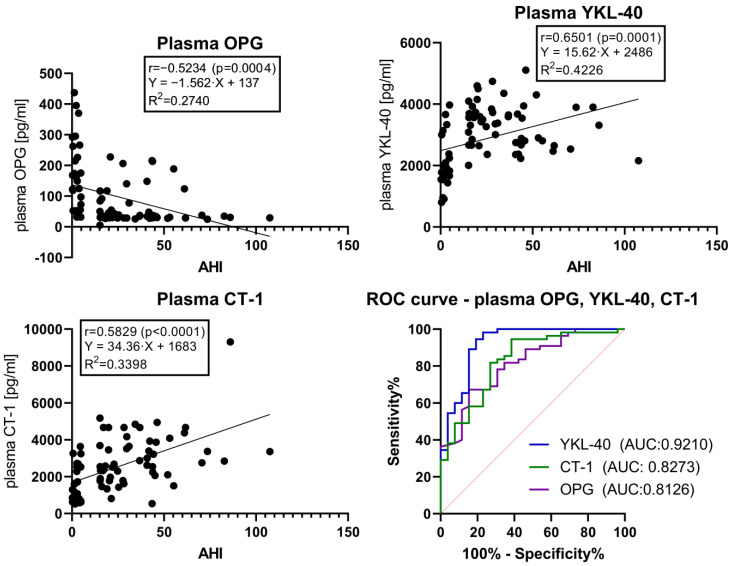
Simple linear regression graphs of the concentrations of OPG, YKL-40, and CT-1 in the plasma. ROC curves of the concentrations of OPG, YKL-40, and CT-1 in the plasma. The results of Spearman correlation analysis are expressed as r values and the level of statistical significance (*p*). The values of r with *p* < 0.05 were considered statistically significant. The coefficient of determination is expressed as R^2^. The simple linear regression models for serum OPG, YKL-40, and CT-1 use AHI as a single explanatory variable. The Receiver Operator Characteristic curve analysis is calculated for all subjects in classifying for OSA and non-OSA groups. Abbreviations: OSA, Obstructive Sleep Apnea; OPG, Osteoprotegerin; YKL-40, Chitinase-3 like protein-1; CT-1, Cardiotrophin-1; AHI, Apnea–Hypopnea Index; ROC, Receiver Operator Characteristic; AUC, Area Under the Curve.

A Receiver Operator Characteristic (ROC) curve analysis was performed to assess the predictive capabilities of the concentrations of serum and plasma OPG, YKL-40, and CT-1 for distinguishing between the OSA group and the control group. Serum OPG (Area Under the Curve (AUC) = 0.7771, *p* < 0.0001, a cut-off value of <47.78 pg/mL), serum YKL-40 (AUC = 0.9734, *p* < 0.0001, cut-off value of >3759 pg/mL), serum CT-1 (AUC = 0.8749, *p* < 0.0001, cut-off value of >3805 pg/mL), plasma OPG (AUC = 0.8126, *p* < 0.0001, a cut-off value of <37.56 pg/mL), plasma YKL-40 (AUC = 0.9210, *p* < 0.0001, cut-off value of >3343 pg/mL), and plasma CT-1 (AUC = 0.8273, *p* < 0.0001, cut-off value of >1749 pg/mL) present high sensitivity and specificity in predicting the presence of moderate and severe OSA (AHI ≥ 15) (Figure 1 and Figure 2).

We calculated results for sensitivity and specificity, as well as Likelihood Ratios (LR), at the presented cut-off values. Positive LR is the ratio of the proportion of patients who have the target condition and test positive to the proportion of patients without the target condition who also test positive [27]. For example, a test with LR 10 will yield positive results ten times as frequently for subjects with OSA than for subjects without this disease. The sensitivity, specificity, and LR for OSA diagnosis of the concentrations of OPG, YKL-40, and CT-1 in the serum and plasma at the presented cut-off values were:

– serum OPG—sensitivity 50.94%, specificity 96.30%, and LR 13.75;
– serum YKL-40—sensitivity 88.68%, specificity 96.30%, and LR 23.94;
– serum CT-1—sensitivity 62.26%, specificity 92.59%, and LR 8.406;
– plasma OPG—sensitivity 56.36%, specificity 88.46%, and LR 4.885;
– plasma YKL-40—sensitivity 54.55%, specificity 96.15%, and LR 14.18;
– plasma CT-1—sensitivity 38.18%, specificity 96.15%, and LR 9.927.

**Figure 3:**
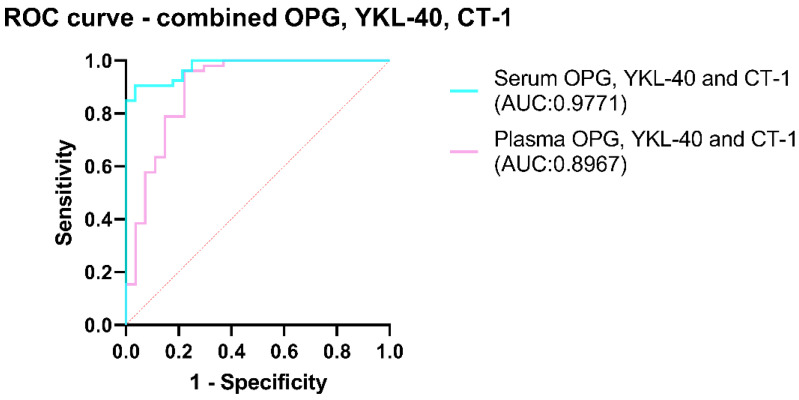
ROC curves of the combined concentrations of OPG, YKL-40, and CT-1 in the serum and plasma. Receiver Operator Characteristic curve analysis is calculated for all subjects in classifying for OSA and non-OSA groups. Abbreviations: OSA, Obstructive Sleep Apnea; OPG, Osteoprotegerin; YKL-40, Chitinase-3 like protein-1; CT-1, Cardiotrophin-1; AHI, Apnea–Hypopnea Index; ROC, Receiver Operator Characteristic; AUC, Area Under the Curve.

A combination of three potential biomarkers—OPG, YKL-40, and CT-1—was performed for predicting classification into OSA and non-OSA subjects. Multiple logistic regression models including the concentrations of OPG, YKL-40, and CT-1 in the serum and plasma showed that only YKL-40 was a non-zero estimate (in the serum, *p*-value = 0.0019; in the plasma, *p*-value = 0.0005), while OPG and CT-1 *p*-values were not significant. The ROC curves of the combined OPG, YKL-40, and CT-1 models are shown in Figure 3.

After adjusting for the subjects’ age, sex, BMI, and AHI, the concentrations of OPG, YKL-40, and CT-1 in the serum and plasma were shown to be dependent on AHI. Plasma YKL-40 was revealed to be inversely dependent on BMI—the higher the BMI, the lower the plasma YKL-40. Calculated *p*-values for multiple linear regression estimates, with age, sex, BMI, and AHI as independent variables, are displayed in Table 4.

### 2.2. TOS, TAS, and OSI

**Figure 4:**
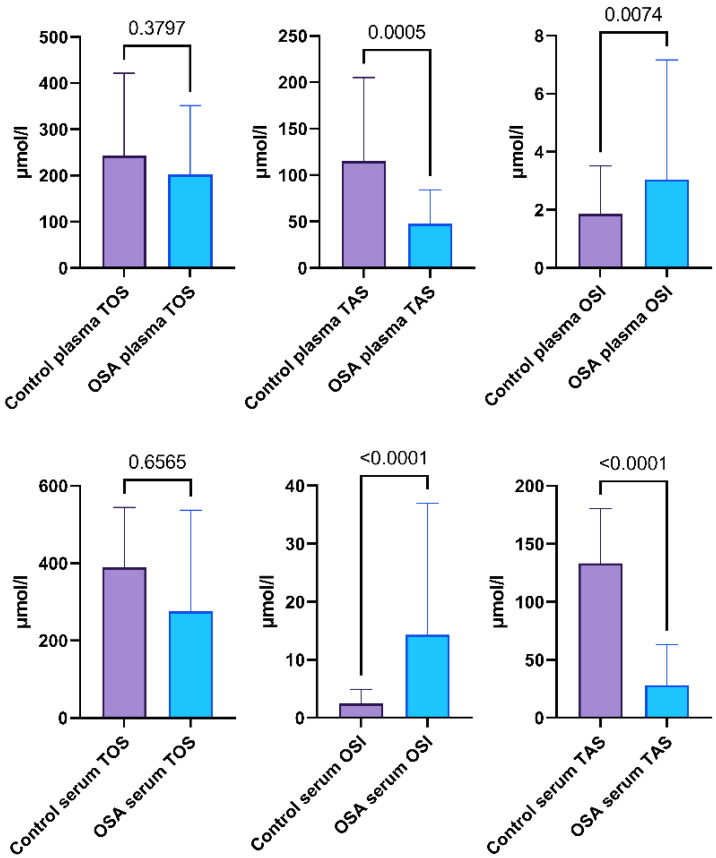
Total oxidative status (TOS), total antioxidative status (TAS), and oxidative stress index (OSI) of the serum and plasma of the participants from the OSA group and control group. Data represent the median and interquartile range and *p*-value of the OSA group vs. the control group.

Subjects with OSA exhibited a 4.7 times lower TAS and 5.8 times higher OSI compared to the non-OSA control group. In the plasma, TAS was 2.4 times lower and OSI was 63% higher when comparing the OSA group to the control group. There was no difference in TOS in the serum and plasma between the OSA and control groups. A visual representation of the results is shown in Figure 4. The calculated medians, minimum-maximum values, and interquartile ranges of TOS, TAS, and OSI in the serum and plasma are demonstrated in Supplementary Material Table S1.

**Figure 5:**
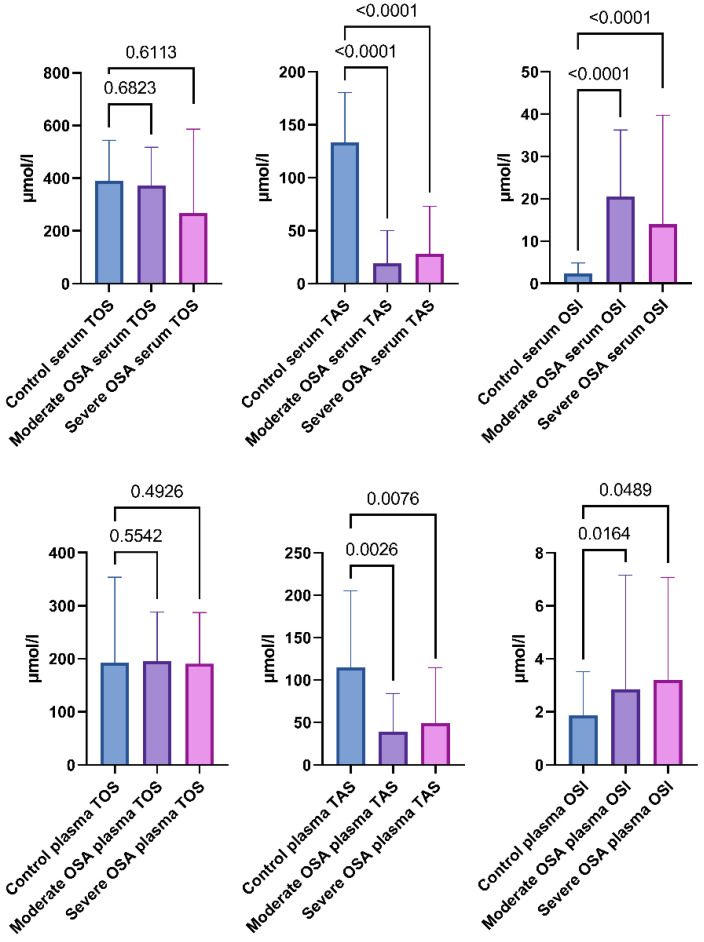
Total Oxidative Status (TOS), Total Antioxidative Status (TAS), and Oxidative Stress Index (OSI) of the serum and plasma of the participants from the Moderate OSA group, Severe OSA group, and control group. Data represent the median and interquartile range and *p*-value of the OSA group vs. the control group.

TOS, TAS, and OSI were established in the Moderate OSA group and the Severe OSA group. Subjects with Moderate OSA exhibit 6.9 times lower TAS and 14.7 times higher OSI compared to the non-OSA control group. In the plasma, TAS was 3 times lower and OSI was 53% higher when comparing the Moderate OSA group to the control group. Subjects from the Severe OSA group show a 4.7 times lower TAS and 5.7 times higher OSI compared to the non-OSA control group. In the plasma, TAS was 2.3 times lower and OSI was 72% higher when comparing the Severe OSA group to the control group. There was no difference in TOS in the serum and plasma between the Moderate and Severe OSA and control groups. A visual representation of the results is shown in Figure 5. The calculated medians, minimum-maximum values, and interquartile ranges of TOS, TAS, and OSI in the serum and plasma are demonstrated in Supplementary Material Table S2.

Numerous positive or negative correlations were noted between the investigated parameters (Table 5). The value of AHI, reflecting the severity of OSA disorders, positively correlated with the age of subjects, BMI, serum and plasma TAS, and serum OSI and with the concentrations of OPG, YKL-40, and CT-1 in the serum and plasma. There were correlations between the studied potential biomarkers and oxidative stress parameters—serum TAS correlated positively with the concentration of OPG and negatively with the concentrations of YKL and CT-1 in serum and plasma. Serum OSI correlated positively with the concentrations of YKL-40 and CT-1 in the serum and plasma. There was no dependence between TOS and the concentrations of OPG, YKL-40, and CT-1 in the serum and plasma.

## 3. Discussion

The present investigation is the first one assessing the concentrations of OPG, YKL-40, and CT-1 of a single cohort of sleep apnea patients compared to non-OSA controls. It is also the first study to evaluate and compare the diagnostic capability of OPG, YKL-40, and CT-1 in both serum and plasma. Moreover, to our knowledge, the present study is the first report showing a dependence between OPG, YKL-40, and CT-1 and oxidative/reductive status in OSA, as measured by decreased TAS and increased OSI. The research provided evidence for OPG, YKL-40, and CT-1 to be considered potential biomarkers for diagnosing moderate and severe OSA.

Results from preliminary studies regarding the concentrations of OPG in OSA subjects in comparison to non-OSA controls are contradictory. A study by Wen et al. reported that concentrations of OPG in the plasma were significantly higher in 120 patients with OSA compared to the 40 subjects from the control group, was associated with the presence of OSA, and correlated with AHI [14]. The study by Ma et al. found lower OPG concentrations in the serum in the OSA group compared to the control group [28]. The study by Kosacka et al. revealed no differences in the concentrations of OPG in the serum between OSA patients and the control group and described no correlations between the concentrations of serum OPG and AHI, although there were higher serum concentrations of OPG between OSA patients with cardiovascular disease (CVD) and OSA patients without CVD [29]. A study by Nizam et al. reported no differences in terms of the concentrations of OPG in serum between non-OSA controls and mild-to-moderate and severe OSA patient groups [30].

YKL-40 has been widely researched as a potential biomarker for OSA [17,18,31,32,33,34,35]. A meta-analysis by Zhang et al. included five studies of varying quality, with patients from different demographic backgrounds and different baseline characteristics, and concluded that OSA patients present elevated serum YKL-40 concentrations, which may serve as a potential biomarker for diagnosis and screening of OSA. However, due to the small number of studies (less than 10), the construction of a funnel plot and assessment of publication bias was impossible. Our study confirms the findings from the meta-analysis, namely, that the concentrations of YKL-40 correlated with BMI (albeit in our study, only in the control group in the concentrations of YKL-40 in plasma). Our study revealed a positive correlation between the concentrations of YKL-40 in the serum and plasma in patients suffering from OSA and may help lay the groundwork for further research regarding the practical usage of YKL-40 as a biomarker.

The association between CT-1 concentrations and OSA remains inconclusive. Hypoxic stress is known to induce cardiotrophin-1 expression in cardiac myocytes through hypoxia-induced factor-1 (HIF-1), which is a part of the cell survival response [36,37]. Cakir et al. found that the concentrations of CT-1 in serum were significantly elevated in OSA subjects and were higher in the severe OSA patient group than those in the mild/moderate patient group [22]. On the other hand, a study by Kurt et al. reported no significant difference in the plasma concentrations of CT-1 between the OSA group and the controls. The plasma concentrations of CT-1 were not different among the three OSA groups—mild, moderate, and severe [23]. Our study provides further incentive to look into the role of CT-1 in diagnosing, screening, or monitoring OSA.

In OSA patients, repeated episodes of hypoxia and reoxygenation that result from apneas and hypopneas lead to oxidative stress, which may disrupt endothelial function [38]. The alterations in the oxidative/antioxidative imbalance cause further tissue dysfunction and damage to the defenses against oxidative stress, as was reflected in decreased TAS and increased OSI in OSA patients.

The increase in the value of OSI in the serum and plasma in the OSA group compared to the control group indicates the disturbance of the balance between the processes of oxidation and reduction, leading to the development of oxidative stress. The fact that in the OSA group, the serum and plasma TAS were markedly decreased, while TOS was unchanged compared to the control group, shows that the main cause of the oxidative stress was the weakening of the antioxidative capacity. Moreover, these processes were more profound in the Moderate OSA group than in the Severe OSA group, as the Moderate OSA group had lower TAS in both serum and plasma than the Severe OSA group. OSI was higher in the Moderate group compared to the control group when measured in serum, but no difference was found in plasma. This may indicate that the severity of OSA, as classified by AHI, is not solely responsible for the extent of oxidative stress in OSA, and other unknown factors may be involved.

TOS, TAS, and OSI exhibited several correlations with OPG, YKL-40, and CT-1. Previously, only one study aimed to link one of the proposed biomarkers, the serum concentration of OPG, with decreased total antioxidative capacity and found no significant dependencies [28].

While analyzing the available literature, it is important to distinguish whether the assessments were performed in the serum or plasma. The studies regarding the divergence between the concentrations of OPG, YKL-40, or CT-1 in the serum and plasma are limited, if not lacking. We chose to include both mediums to analyze which provide a better diagnostic capability for moderate and severe OSA. The concentrations of OPG, YKL-40, and CT-1 in the plasma correlated with AHI more closely than the concentrations of OPG, YKL-40, and CT-1 in the serum. Our results indicate that for estimating the severity of the disease using the concentrations of OPG, YKL-40, or CT-1, the concentrations in the plasma and not in the serum should be used.

The ROC curve analysis performed for the concentrations of YKL-40 and CT-1 in the serum and plasma showed more accurate predictive capabilities for the concentrations of YKL-40 and CT-1 in the serum compared to plasma. Contrarily, the concentrations of OPG in plasma displayed a higher ROC AUC value than the concentrations of OPG in the serum. For moderate to severe OSA prediction, i.e., as a screening test, the concentrations of YKL and CT-1 in serum and the concentrations of OPG in plasma could be utilized.

There were higher concentrations of OPG, YKL-40, or CT-1 in the serum compared to the concentrations of OPG, YKL-40, or CT-1 in the plasma. This may be caused by the serum or plasma preparation procedure, alterations of the results by the anticoagulating agent present in plasma samples, or an unknown factor. Additional studies could potentially investigate the cause of the disparity between individual subjects’ concentrations of OPG, YKL-40, or CT-1 in the serum and plasma.

Further studies may inquire whether the concentrations of OPG, YKL-40, and CT-1 in the serum and plasma normalize after standard treatment, i.e., PAP therapy. This would verify the feasibility of using OPG, YKL-40, and CT-1 as biomarkers for monitoring the effectiveness of treatment in OSA.

De Luca Canto et al. found that several individual OSA serum and plasma biomarkers, such as combined kallikrein-1, uromodulin, urocortin-3, and orosomucoid-1 in children and IL-6 and IL-10 plasma concentrations in adults, have adequate diagnostic utility for identifying or excluding the presence of OSA [12]. Alterations in the concentrations of the aforementioned compounds are theorized to be caused by OSA-induced chronic inflammation, hypoxemia, sleep fragmentation, and endothelial and metabolic dysfunctions. A recent 2022 systematic review by Gaspar et al., building upon the work of De Luca Canto et al., after evaluating 16 studies concerning 2156 individuals, found that the most promising candidates for biomarkers for OSA diagnosis were Endocan and YKL-40 in the serum, Interleukin-6 and Vimentin in the plasma, and mRNA concentrations of a disintegrin and metalloprotease domain 29 (ADAM29), Fibronectin Leucine Rich Transmembrane Protein 2 (FLRT2), and Solute Carrier Family 18 Member A3(SLC18A3) in human peripheral blood mononuclear cells (PBMCs) [31]. Unfortunately, the diagnostic capability of different studied biomarkers or combinations of biomarkers is still inconclusive in identifying OSA or accurately predicting the disease burden.

### Study Limitations

There are several limitations to this study.

First, because of the relatively small number of participants (less than 100), conclusions should be drawn with care. However, effect size statistics suggest that the number of participants was large enough to perform the statistical analysis and establish conclusions.

The patients did not have thorough polysomnography to diagnose OSA. All patients had a type-3 sleep study that measured four physiologic variables, including two respiratory variables (respiratory movement and airflow), a cardiac variable (heart rate), and arterial oxygen saturation, as recommended by the Clinical Practice Guideline for Diagnostic Testing for Adult Obstructive Sleep Apnea [2]. The polygraph test also offers benefits, such as the fact that it was administered at home rather than in a hospital, increasing the likelihood that the subject would sleep without disturbances. The report was not created automatically. The first and second authors, in each case, manually read the sleep record to get the report. 

The study did not include mild OSA patients, which may limit the utility of our results for certain potential biomarkers for screening and diagnosis of OSA in every stage. Regarding this, mild OSA patients have substantially less severe symptoms and are less likely to be admitted to the hospital where the study was conducted, making it difficult to establish a sufficiently sized mild apnea group for this study. We decided to not include the mild OSA patients in this study due to a limited number of subjects, which was insufficient to establish the study group and perform statistical analysis.

The control group was marginally significant in terms of age and sex and different from the OSA group in terms of BMI. Despite that, possible confounding factors, such as BMI, age, and sex, did not correlate with the concentrations of studied compounds in OSA patients, except for BMI in comparison to the concentration of YLK-40 in the plasma. Although it would be better to compare the study group to a perfectly matched control group, this difference should not impact the study’s overall results.

## 4. Materials and Methods

### 4.1. Ethical Approval and Informed Consent

The study was approved by the Bioethics Committee of the Medical University of Bialystok (Poland; approval no. APK.002.207.2020) and conducted according to GCP/Guidelines for Good Clinical Practice. A signed written informed consent form and acceptance to participate in the study were received from all participants of the study.

### 4.2. Study Protocol

The research was carried out at a University Hospital in Bialystok’s Department of Otolaryngology between September 2020 and October 2022. Subjects were accepted or rejected depending on inclusion and exclusion criteria.

The inclusion criteria for the OSA group were: age 18 years < age ≤ 65 years, polygraph-documented moderate (15 ≤ AHI ˂ 30) and severe (AHI ≥ 30) OSA, and signed written informed consent. 

We excluded patients with central sleep apnea syndrome; morbid obesity (BMI ≥ 40); immune-suppression therapy with inhaled, oral, or nasal steroids or other anti-inflammatory drugs within 3 months before enrollment; treatment for sleep apnea within the 3 months before enrollment; history of respiratory infection within the previous 4 weeks; history of rheumatic diseases, coagulation disorders, or acute or chronic kidney failure; history of injury or surgery in the past 3 months; severe cardiovascular disease; systemic inflammatory diseases; and comorbidities that may affect systemic inflammation, such as collagen vascular disease or cancer, chronic rhinosinusitis, administration of hormones, immune suppressors, or free radical scavengers.

The control group was composed of non-OSA adults with no symptoms of OSA, no snoring, no local or systemic inflammatory disease, and AHI < 5 (confirmed by sleep study type III polygraph (PG).

All patients underwent the same procedures, according to the study protocol: a medical history, BMI, otorhinolaryngological examination, and PG.

### 4.3. Sleep Study

A PG was performed in each case using a type III sleep study device (SOMNOtouch, SOMNOmed). During the PG, the following parameters were evaluated for this study: AHI, mean oxygen saturation (MOS) and lowest oxygen saturation (LSAT), and time of sleep spent with blood oxygen saturation less than 90% (SpO_2_ < 90).

The AHI is described as the total number of apnea and hypopnea events per hour of sleep recorded in an overnight sleep study. Apneas are defined as at least a 90% decrease in airflow for at least 10 s and hypopneas as a reduction of respiratory signals for at least 10 s associated with a minimum of 3% of oxygen desaturation. MOS is estimated as normal and varies between 94% and 98% during sleep [2].

### 4.4. Blood Sampling

The whole blood was collected from the subjects to obtain the serum and plasma for the evaluation of the concentrations of OPG, YKL-40, and CT-1. A fasting blood sample was gathered in the morning between 7–9 a.m. Blood for plasma samples was collected into two 2.7 mL tubes treated with the anticoagulant, ethylenediaminetetraacetic acid (EDTA), and was centrifuged immediately after collection. Blood for serum samples was collected into two 5.5 mL tubes with plastic granules with a coagulation activator and allowed to completely clot for 30 min at room temperature. A total of approximately 16.4 mL of venous blood was centrifuged at 2000× *g* for 10 min at +4 °C, and obtained serum and plasma were stored at −80 °C until the assays were performed. 

### 4.5. Serum and Plasma Laboratory Testing

OPG, YKL-40, and CT-1 concentrations in the serum and plasma were determined using commercially available human enzyme-linked immunosorbent assay (ELISA) kits by Biorbyt (Cambridge, UK)—Biorbyt Human OPG ELISA Kit (orb50091), Biorbyt Human YKL-40 ELISA Kit (orb219580), and Biorbyt Human Cardiotrophin-1 ELISA kit (orb219241). The analyses were performed strictly following the manufacturer’s instructions. Measurement of the concentrations of OPG, YKL-40, and CT-1 was carried out in the same laboratory, by the same person. The quantification of investigated parameters was performed with the use of the ELISA universal microplate reader Epoch (BioTek Instruments, Inc., Winooski, VT, USA). The precision of measurement was expressed as a coefficient of variation (CV). The intra-assay CV was <1.8%, 4.7%, and 6% for OPG, YKL-40, and CT-1, respectively, whereas the inter-assay CV was <2.88%, 1.2%, and 3.3%, respectively.

TOS and TAS were determined in duplicate using commercially available enzyme-linked immunosorbent assay (ELISA) kits by Immundiagnostik AG (Bensheim, Germany). The analyses were performed strictly following the manufacturer’s instructions. TOS was measured in the serum and plasma using the PerOx (TOS) Kit, based on the determination of the total lipid peroxides present in the investigated sample in the reaction with peroxidase at 450 nm. TAS was determined using the ImAnOx (TAS) ELISA Kit, based on the reaction of the elimination of hydrogen peroxide added into the investigated sample by antioxidants present in the sample. The residual hydrogen peroxide generates products that absorb at 450 nm. The quantification of investigated parameters was performed with the use of the ELISA universal microplate reader Epoch (BioTek Instruments, Inc., Winooski, VT, USA).

The analytical quality of these assays was checked by the measurement of particular parameters in control samples included in the kits and the calculation of the intra- and inter-assay coefficient of variation (CV). The values of TOS and TAS determined by us in the control samples included in the kits agreed with the certified values. The intra-assay CVs were <3% and <7% for TOS and TAS, respectively, whereas the inter-assay CVs were <3% and <2%, respectively. The quality control of TOS and TAS measurements confirmed the reliability of the obtained results. The severity of oxidative stress was estimated based on the value of OSI, calculated as the ratio of TOS and TAS (OSI = TOS/TAS).

### 4.6. Statistical Analysis

The statistical analysis was performed using GraphPad Prism version 9.0.0 for Windows (GraphPad Software, San Diego, CA, USA). Data were first tested for normal distribution using the Shapiro–Wilk test. If there was no normal distribution of the data, a nonparametric Mann–Whitney’s U test was conducted for comparisons between the groups. A multiple linear regression model was used to assess confounders for AHI in different groups. To estimate mutual dependencies between AHI and the concentrations of serum and plasma OPG, YKL-40, and CT-1, Spearman correlation analysis was performed. The Cohen’s d and effect size r statistic was used to measure the effect sizes for the difference (the strength of the difference) in the values of the concentrations of OPG, YKL-40, and CT-1 in the serum and plasma in all subjects, namely, the control group, the Moderate OSA group, and the Severe OSA group. Receiver operating characteristic (ROC) curve analysis was performed to determine the area under the curve for the sensitivity and specificity of OPG, YKL-40, and CT-1 in distinguishing between non-OSA controls and moderate-to-severe OSA subjects. All statistical hypotheses were verified at a significance level of α = 0.05

## 5. Conclusions

YKL-40, CT-1, and OPG are useful candidates as diagnostic biomarkers for moderate and severe OSA. The concentrations of YKL-40 and CT-1 in serum and OPG in plasma show better diagnostic capabilities for moderate and severe OSA than the concentrations of YKL-40 and CT-1 in plasma and OPG in serum. It is suggested that for the severity of OSA, the evaluation of the concentrations of OPG, YKLl-40, and CT-1 in plasma is more useful than those in serum.

## Figures and Tables

**Table 1:** Demographic, anthropometric, and biological characteristics of the OSA and non-OSA participants of the study.

	OSA Group (n = 52)	Control Group (n = 28)	p*	Moderate OSA Group (n = 27)	*p*^	Severe OSA Group (n = 25)	*p*”
**Sex male/female** **(% of male)**	44/8 (85%)	18/10 (64%)	0.1001	23/4 (82%)	0.2270	21/4 (84%)	0.1286
**Age [years]**	41; 21–67 (IQR 35–55)	37; 24–74 (IQR 29–52)	0.0628	37; 20–65 (IQR 34–53.50)	0.5006	45.76 ± 12.81	0.0761
**BMI [kg/m^2^]**	28.62; 21.02–39.31 (IQR 26.86–30.67)	26.57 ± 2.948	0.0022	28.67 ± 2.847	0.0147	31.46 ± 4.663	0.0002
**AHI [events/hour]**	28.30; 15.40–107.5 (IQR 19.90–43.90)	2.45; 0.1–4.9 (IQR 0.575–3.975)	<0.0001	19.45; 15.40–29.80 (IQR 15.90–24.33)	<0.0001	45.10; 31–107.5 (IQR 41.35–61.40)	<0.0001
**MOS [%]**	93; 70–96 (IQR 91.3–94)	96; 93–97 (IQR 95–97)	<0.0001	94; 88–96 (IQR 92.13–95)	<0.0001	93; 70–96 (IQR 90.5–93)	<0.0001
**LSAT [%]**	81; 35–92 (IQR 75–84)	92.5; 86–95 (IQR 91–93.25)	<0.0001	82.57 ± 5.014	<0.0001	75; 35–87 (IQR 65–82)	<0.0001
**SpO_2_ < 90% [%]**	5.9; 0–84.5 (IQR 1.2–25.7)	0; 0–0.2 (IQR 0–0)	<0.0001	2.1; 0–75.9 (IQR 0.325–8.325)	<0.0001	24.4; 0.6–84.5 (IQR 9.8–41.5)	<0.0001
**Hypertension** **[n (%)]**	23 (44%)	6 (21%)	0.0531	6 (21%)	0.9531	17 (68%)	0.0009
**Comorbidities** **[n (%)]**	9 (17%)	2 (7%)	0.3123	3 (11%)	0.6695	6 (24%)	0.1288
**Tobacco smokers** **[n (%)]**	15 (29%)	5 (18%)	0.4174	4 (14%)	0.7726	11 (44%)	0.0706

Values are presented as mean ± standard deviation—SD (in the case of normal distribution of the data), median and minimum-maximum (Interquartile range) (if there was no normal distribution of the data), or the number of subjects (percentage of the group). Abbreviations: OSA, Obstructive Sleep Apnea; BMI, Body Mass Index; AHI, Apnea–Hypopnea Index; MOS, mean oxygen saturation; LSAT, lowest oxygen saturation; SpO_2_ < 90%, percentage of sleep spent with blood saturation < 90%; p*, *p*-value of the OSA group vs. the control group; *p*^, *p*-value of the moderate OSA group vs. the control group; *p*”, *p*-value of the Severe OSA group vs. the control group; IQR, Interquartile range; n, number of participants.

**Table 2:** The concentrations of OPG, YKL-40, and CT-1 in the serum and plasma.

		OSA Group (n = 52)	Control Group (n = 28)	*p*-Value	Cohen’s *d*	Effect Size *r*
**Serum**	**OPG [pg/mL]**	40.3; 22.2–247 (IQR 27.6–161)	178; 47.5–407 (IQR 70.3–287)	<0.0001	−0.934	−0.423
	**YKL-40 [pg/mL]**	4332 ± 619.2	1905; 1153–3869 (IQR 1729–3269)	<0.0001	2.65	0.798
	**CT-1 [pg/mL]**	4420, 1816–8586 (IQR 2422–5046)	1195; 350.9–4885 (IQR 921.6–2656)	<0.0001	1.609	0.627
**Plasma**	**OPG [pg/mL]**	34.86; 5.355–228 (IQR 29.45–84.23)	155.7; 31.85–437.4 (IQR 52.88–263.4)	<0.0001	−1.090	−0.479
	**YKL-40 [pg/mL]**	3358 ± 710.5	1785; 802.3–3661 (IQR 1552–2132)	<0.0001	2.040	0.714
	**CT-1 [pg/mL]**	2679; 536.7–9308 (IQR 1895–3867)	1017; 524.8–3634 (IQR 757.6–2345)	<0.0001	1.236	0.526

Values are presented as mean ± SD (in the case of normal distribution of the data) or as median and minimum-maximum (Interquartile range) (if there was no normal distribution of the data). Abbreviations: OSA, Obstructive Sleep Apnea; OPG, Osteoprotegerin; YKL-40, Chitinase-3 like protein-1; CT-1, Cardiotrophin-1; IQR, Interquartile range.

**Table 3:** Spearman’s correlation coefficients between AHI and the concentrations of OPG, YKL-40, and CT-1 in the serum and plasma.

		All Subjects (n = 80)		Control Group (n = 28)		Moderate OSA Group (n = 27)		Severe OSA Group (n = 25)	
		AHI Correlation							
		r	*p*-Value	r	*p*-Value	r	*p*-Value	r	*p*-Value
**Serum**	**OPG [pg/mL]**	−0.5022	<0.0001	−0.1589	0.4194	−0.1457	0.4683	0.085	0.6862
	**YKL-40 [pg/mL]**	0.5144	<0.0001	0.1468	0.4559	0.051	0.8006	−0.2950	0.1523
	**CT-1 [pg/mL]**	0.5571	<0.0001	0.07712	0.6965	0.02153	0.9151	0.2505	0.2272
**Plasma**	**OPG [pg/mL]**	−0.5234	<0.0001	−0.2442	0.2196	−0.06614	0.7482	−0.0524	0.8036
	**YKL-40 [pg/mL]**	0.6501	<0.0001	0.426	0.0267	0.1604	0.4337	0.08394	0.6899
	**CT-1 [pg/mL]**	0.5829	<0.0001	0.1176	0.5589	−0.06298	0.7599	0.02388	0.9098

The results of Spearman correlation analysis are expressed as r values and the level of statistical significance (*p*). The values of r with *p* < 0.05 were considered statistically significant. Abbreviations: AHI, Apnea–Hypopnea Index; OSA, Obstructive Sleep Apnea; OPG, Osteoprotegerin; YKL-40, Chitinase-3 like protein-1; CT-1, Cardiotrophin-1.

**Table 4:** Multiple linear regression estimates using sex, AHI, age, and BMI as independent variables.

		Sex *r*^2^ *p*-Value	AHI *r*^2^ *p*-Value	Age *r*^2^ *p*-Value	BMI *r*^2^ *p*-Value
**Serum**	**OPG**	0.4057	0.0022	0.0345	0.4788
	**YKL-40**	0.8957	<0.0001	0.5236	0.4102
	**CT-1**	0.4471	<0.0001	0.7671	0.0545
**Plasma**	**OPG**	0.9654	0.0044	0.1112	0.5041
	**YKL-40**	0.4717	0.0006	0.1001	0.0383
	**CT-1**	0.4839	<0.0001	0.9233	0.9792

The values of r with *p* < 0.05 were considered statistically significant. The multiple linear regression models for serum and plasma OPG, YKL-40, and CT-1 use sex, AHI, age, and BMI as estimates. Abbreviations: OSA, Obstructive Sleep Apnea; OPG, Osteoprotegerin; YKL-40, Chitinase-3 like protein-1; CT-1, Cardiotrophin-1; AHI, Apnea–Hypopnea Index; BMI, Body Mass Index.

**Table 5:** Mutual dependences between the investigated parameters.

	AHI	Age	BMI	Serum TOS	Serum TAS	Serum OSI	Plasma TOS	Plasma TAS	Plasma OSI	Serum OPG	Serum YKL-40	Serum CT-1	Plasma OPG	Plasma YKL-40
Age	0.283 *													
BMI	0.410 ^‡^	0.334 *												
Serum TOS	NS	NS	NS											
Serum TAS	−0.484 ^‡^	NS	NS	0.235 *										
Serum OSI	0.463 ^‡^	0.296 ^‡^	NS	0.296 *	−0.811									
Plasma TOS	NS	NS	NS	0.383	NS	NS								
Plasma TAS	−0.281 *	0.237 *	NS	0.295 *	0.247 *	NS	0.382 ^‡^							
Plasma OSI	NS	NS	NS	NS	NS	NS	0.494 ^‡^	−0.565 ^‡^						
Serum OPG	−0.538 ^‡^	−0.257 *	−0.243 *	NS	0.290 *	NS	NS	NS	NS					
Serum YKL-40	0.662 ^‡^	NS	0.281 *	NS	−0.424 ^‡^	0.368 ^‡^	NS	NS	NS	−0.562 ^‡^				
Serum CT-1	0.593 ^‡^	NS	NS	NS	−0.439 ^‡^	0.388 ^‡^	NS	NS	NS	−0.419 ^‡^	0.566 ^‡^			
Plasma OPG	−0.502 ^‡^	−0.330 *	−0.298 *	NS	0.289 *	NS	NS	NS	NS	0.757 ^‡^	−0.663 ^‡^	−0.480 ^‡^		
Plasma YKL-40	0.514 ^‡^	NS	NS	NS	−0.423 ^‡^	0.308 *	NS	NS	NS	−0.481 ^‡^	0.611 ^‡^	0.453 ^‡^	−0.568 ^‡^	
Plasma CT-1	0.557 ^‡^	NS	NS	NS	−0.327 *	0.353 *	NS	NS	NS	−0.579 ^‡^	0.574 ^‡^	0.737 ^‡^	−0.615 ^‡^	0.495 ^‡^

The results of Spearman correlation analysis are expressed as r values and the level of statistical significance (*p*). The values of r with *p* < 0.05 were considered statistically significant (* *p* < 0.05, ^‡^ *p* < 0.001). Grey background color marks correlations between the corresponding characteristics. NS—not statistically significant (*p* > 0.05). BMI, body mass index; AHI, Apnea–Hypopnea Index; TOS, Total Oxidative Status; TAS, Total Antioxidative Status; OSI, Oxidative Stress Index; OPG, Osteoprotegerin; YKL-40, Chitinase-3 like protein-1; CT-1, Cardiotrophin-1.

## Data Availability

The data supporting this study’s findings are available on request from the corresponding author.

## Supplementary Materials

The following supporting information can be downloaded at: https://www.mdpi.com/article/10.3390/ijms24032607/s1.
