# Supplementary material for: Osteoprotegerin, Chitinase 3-like Protein 1, and Cardiotrophin-1 as Potential Biomarkers of Obstructive Sleep Apnea in Adults—A Case-Control Study
Source: Int J Mol Sci. 2023 Jan 30;24(3):2607. doi: 10.3390/ijms24032607 (PMC9916736; doi:10.3390/ijms24032607)
Supplement: Supplementary file 1 [file ijms-24-02607-s001.zip › ijms-2097313-supplementary.pdf]

Supplementary Material

Osteoprotegerin, Chitinase 3-like Protein 1, and Cardiotrophin-1 as Potential Biomarkers of Obstructive Sleep Apnea in Adults—A Case-Control Study

Piotr Fiedorczuk [1,*], Ewa Olszewska [1], Joanna Rogalska [2] and Małgorzata M. Brzóska [2]

[1] Department of Otolaryngology, Medical University of Bialystok, 15-089 Bialystok, Poland
[2] Department of Toxicology, Medical University of Bialystok, 15-089 Bialystok, Poland
* Correspondence: piotr.fiedorczuk@sd.umb.edu.pl; Tel.: +48-663-751-516

Supplementary Material - Table S1 – Total Oxidative Status (TOS), Total Antioxidative Status (TAS), and Oxidative Stress Index (OSI) of the serum and plasma

		OSA group (n=52)	Control group (n=28)	p-value
**Serum**	TOS	275.9; 19.26 - 1352 (IQR 153.0- 537.4)	389.7; 98.31 - 1037 (IQR 154.5- 544.4)	0.6565
	TAS	28.28; 0.7300 - 283.9 (IQR 5.973 - 63.03)	133.2; 34.87 - 345.7 (IQR 85.52 - 180.3)	**<0.0001**
	OSI	14.31; 0.4952 - 246.5 (IQR 5.221 - 36.94)	2.468; 0.7322 - 13.08) (IQR 1.600 - 4.874	**<0.0001**
**Plasma**	TOS	185.5; 8.110 - 724.6. (IQR 77.03 - 281.7	192.1; 16.35 - 678.4 (IQR 107.9 - 353.9)	0.3797
	TAS	47.80; 1.100 - 226.2 (IQR 27.51 - 84.10)	115.1; 19.77 - 309.2 (IQR 47.50 - 205.1)	**0.0005**
	OSI	3.045; 0.2270 - 103.4 (IQR 1.492 - 7.173)	1.860; 0.000 - 5.920 (IQR 0.8675 - 3.510)	**0.0074**

Values are presented as median and minimum-maximum (Interquartile range).

Abbreviations: OSA, Obstructive Sleep Apnea; TOS, Total Oxidative Status; TAS, Total Antioxidative Status; OSI, Oxidative Stress Index; IQR, Interquartile range

Supplementary Material - Table S2 – Total Oxidative Status (TOS), Total Antioxidative Status (TAS), and Oxidative Stress Index (OSI) of the serum and plasma of Moderate and Severe OSA groups.

		Control group (n=28)	Moderate OSA group (n=27)	p-value compared to the Control group	Severe OSA group group (n=25)	p-value compared to the Control group
**Serum**	TOS	389.7; 98.31 - 1037 (IQR 154.5- 544.4)	372.0; 19.26 - 989.2 (IQR 135.8 - 517.9)	0.6823	268.6; 36.49 -1352 (IQR 160.0 - 586.0)	0.6113
	TAS	133.2; 34.87 - 345.7 (IQR 85.52 - 180.3)	19.22; 1.990 - 148.1 (IQR 5.110 - 49.99)	<0.0001	28.46; 0.7300 - 283.9 (IQR 7.115 - 72.90)	<0.0001
	OSI	2.468; 0.7322 - 13.08) (IQR 1.600 - 4.874	36.29; 0.6854 - 246.5 (IQR 7.496 - 20.59)	<0.0001	13.97; 0.4952 - 166.0 (IRQ 6.095 - 39.74)	<0.0001
**Plasma**	TOS	192.1; 16.35 - 678.4 (IQR 107.9 - 353.9)	195.1; 8.110 - 563.2 (IQR 96.45 - 288.5)	0.5542	190.5; 39.53 - 724.6 (IQR 77.03 - 287.1)	0.4926
	TAS	115.1; 19.77 - 309.2 (IQR 47.50 - 205.1)	38.61; 5.770 - 226.2 (IQR 26.77 - 84.01)	0.0026	49.26; 1.100 - 157.3 (IQR 28.10 - 114.8)	0.0076
	OSI	1.860; 0.000 - 5.920 (IQR 0.8675 - 3.510)	2.851; 0.2270 - 50.89 (IQR 1.492 - 7.162)	0.0164	3.212; 0.3100 - 103.4 (IQR 1.459 - 7.076)	0.0489

Values are presented as median and minimum-maximum (Interquartile range).

Abbreviations: OSA. Obstructive Sleep Apnea; TOS. Total Oxidative Status; TAS. Total Antioxidative Status; OSI. Oxidative Stress Index; IQR. Interquartile range
